# Stump appendicitis: a myth that can become reality

**DOI:** 10.11604/pamj.2020.36.274.24690

**Published:** 2020-08-12

**Authors:** Atef Mejri, Khaoula Arfaoui, Badreddine Aloui

**Affiliations:** 1 Service de Chirurgie Viscérale et Digestive, Hôpital Régional de Jendouba, Jendouba, Tunisie

**Keywords:** Apendectomy, laparoscopy, emergency, complication

## Abstract

Stump appendicitis is a rare etiology of acute lower right quadrant abdominal pain often forgotten in the emergency room (ER). The Mac Burney scar or a previous laparoscopic appendectomy always rule out the eventuality of appendicitis and mislead management. Advanced imaging tools are more than compulsory to help correct the diagnosis. Computed tomography (CT) scan is the option of choice that may be replaced if unavailable by simple ultrasound examination. The treatment is mainly surgical. We report the case of a stump appendicitis occurring 12 years after laparoscopic appendectomy in an 18-year-old girl. The diagnosis was based on consistent clinical signs and conclusive radiological data. A successful completion appendectomy was performed with good outcome.

## Introduction

Appendectomy is the most common surgical procedure in emergency abdominal surgery. Stump appendicitis is a rare complication after appendectomy. Its clinical presentation is comparable to that of acute appendicitis. Although unusual, it should be included in the differential diagnosis of right lower quadrant pain in patients who have already had an appendectomy. Recognition of this entity is crucial because a delayed diagnosis can cause serious complications. We report a case of stump appendicitis in an 18-year-old girl who underwent laparoscopic appendectomy at the age of six years.

## Patient and observation

**Figure 1:**
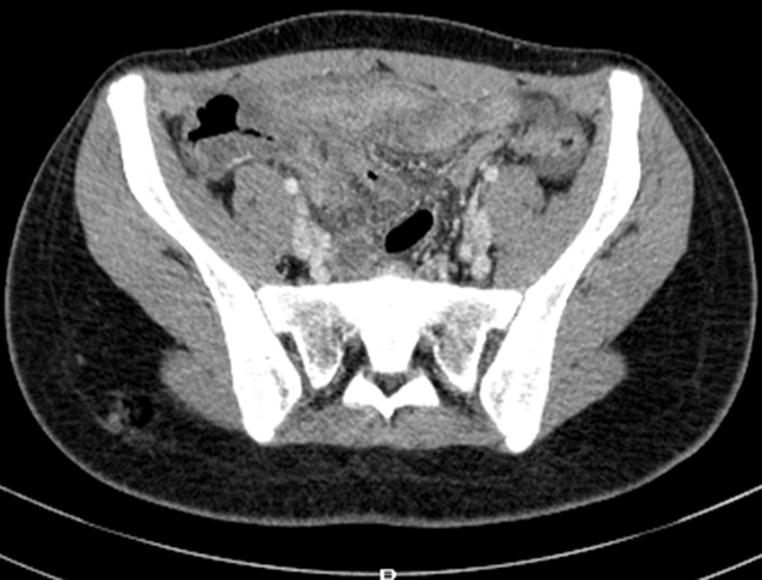
an axial CT scan of abdomen showing inflammatory features surrounding a blind-ending structure arising from the caecum

**Figure 2:**
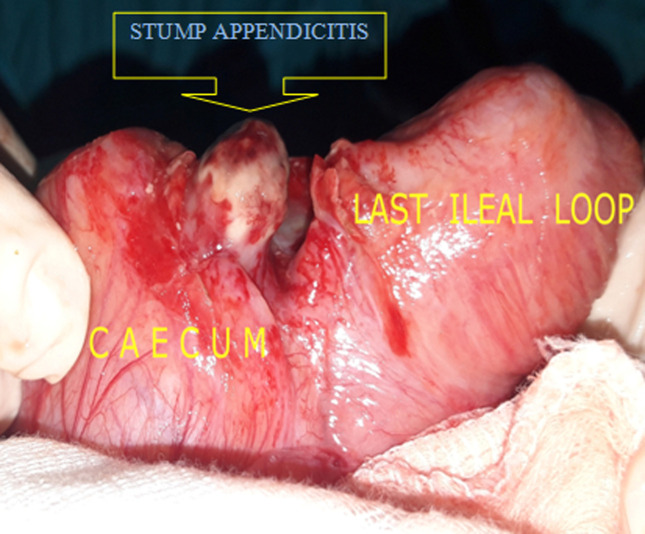
an intraoperative view showing an inflamed appendiceal stamp

An 18-year-old female patient presented to the emergency room for pain in the right iliac fossa evolving for 24 hours with nausea without associated fever, gastrointestinal motility disorders or urinary tract infection symptoms. The patient underwent a laparoscopic appendectomy at the age of 6 years and was operated on for acute bowel obstruction due to adhesions via a midline incision at the age of 12 years. On examination, body temperature was 37.6°C; the hemodynamic parameters were within normal limits. The blood pressure was 112/64, the heart rate 85 bpm. Physical examination reveals rebound tenderness in the right iliac fossa. The rest of the abdomen was soft and non tender. The routine blood tests showed a biological inflammatory syndrome with a white blood cells count of 12, 5 x 10^9^/L and a C-reactive protein rate of 80mg/L. An abdominal ultrasound showed a hypoechoic thickened tubular structure of more than 8mm diameter in the right iliac fossa. An abdominal CT scan showed pericoecal inflammatory change with an enhancing wall tubular structure extending from the internal side of the caecum suspect of appendiceal remnant, associated with a mild amount of free fluid (Figure 1). The preoperative diagnosis of stump appendicitis was then strongly suggested. The patient underwent emergency surgical intervention using the previous lower midline incision. An approximately 1cm long inflamed appendiceal stump was found with pericoecal inflammatory features (Figure 2). A completion appendectomy was performed with good outcome and the patient was discharged on post-operative day 3. The anatomopathological examination of the resected specimen confirmed the diagnosis of suppurative stamp appendicitis.

## Discussion

Considered as a delayed complication of a very common surgical procedure performed worldwide which is conventional appendectomy, stump appendicitis is a rare pathology with an incidental rate of 1/50000 [1]. It is defined as an inflammation of the residual appendix tissue due to incomplete removal of the appendix. This complication occurs particularly on appendicular stumps larger than 5mm, allowing the reconstitution of faecoliths that cause inflammation [2]. The anatomical positional factors include a retro-coecal appendix, subserosal appendix and duplicated appendix. All these situations make it difficult to identify the coecal appendicular junction and contribute to this complication [1]. Moreover, the technical difficulties related to the absence of the clear dimensional vision and the digital palpation in laparoscopy increase the risk of failing to staple the appendix at its base flush with the caecum, leaving behind a large stump that can lead to a recurrence. However, this complication is most often reported after laparotomy [3]. Stump appendicitis is a rare etiology of abdominal pain, often mimicking other causes of acute pain in the lower right quadrant of the abdomen. The most common symptoms are pain or tenderness in the right iliac fossa. Nausea and vomiting and sometimes fever can be noticed. The clinical presentation is very similar to that of acute appendicitis [3].

This condition is often not considered as a preliminary differential diagnosis by clinicians during the first physical examination because of the medical history of previous appendectomy, hence the diagnostic challenge. Radiological examinations are of a great value in this particular case since they help establish a diagnosis already ruled out by the simple presence of a Mc Burney incision scar. The ultrasound signs are similar to those found in the usual acute appendicitis. These include the presence of a noncompressible dilated and wall thickened tubular structure (diameter >6mm), arising from the cecum, with peri appendiceal inflammatory reaction. A hyperechoic small formation may also indicate the presence of a foecolith [4,5]. Computed tomography of the abdomen (CT) is more contributive than ultrasound for an accurate diagnosis of stump appendicitis [6]. CT scan findings include pericoecal inflammatory changes and fat stranding, thickening of the cecum wall and terminal ileum, an ileo-cecal mass, the presence of an abscess and in the pericoecal region and right para-colic gutter [1,5,7]. The arrowhead sign refers to the focal coecal thickening centered on the appendicular opening, considered as a secondary sign in acute appendicitis and stump appendicitis. In cases of persistent ambiguity, diagnostic laparoscopy is the best option [1].

The treatment of choice for appendicitis on stumps is completion appendectomy either by the conventional laparotomy or by laparoscopy. An ileo-coecal resection may be necessary in case of extensive inflammation, peritonitis and/or perforation [4,7]. There is no consensus on which surgical approach to choose. In the stump appendicitis series of the retrospective study of Dikicier *et al*. conducted on 3130 patients who underwent appendectomy between 2008 and 2017, three patients were successfully managed via open Mac Burney laparotomy. Laparoscopy was performed for two patients primarily for diagnostic purpose and conversion was necessary for one of them [7]. A literature review has shown that more than half of the reported stump appendicitis required conventional appendectomy and almost a third of the cases underwent major bowel resection [4]. In order to minimize the risk of this pathology, it is admitted that no appendiceal stump larger than 3mm should be left behind during the first appendectomy [1]. This can be ensured only by a good identification of the appendicular base by following the coecal taenia coli and a complete dissection of any subsurosal appendix [2]. The contribution of inversion remains controversial among surgeons and there is no clear evidence of the superiority of this surgical procedure in the prevention of stump appendicitis compared to simple ligation [6].

## Conclusion

The non-specific clinical presentation and especially the history of an appendectomy can make the diagnosis of stump appendicitis a challenge for the unfamiliarized practitioner and lead to a therapeutic delay with serious consequences. Although rare, this diagnosis must therefore be kept in mind in every case of any pain in the right iliac fossa. Radiology plays a key role in eliminating other differential diagnoses and dispelling any diagnostic doubts. The treatment is above all surgical.
